# Mechanisms and Treatment Options for Hyperthyroid-Induced Osteoporosis: A Narrative Review

**DOI:** 10.7759/cureus.48798

**Published:** 2023-11-14

**Authors:** Robert M Branstetter, Rahib K Islam, Collin A Toups, Amanda N Parra, Zachary Lee, Shahab Ahmadzadeh, Giustino Varrassi, Sahar Shekoohi, Alan D Kaye

**Affiliations:** 1 School of Medicine, Louisiana State University Health School of Medicine, New Orleans, USA; 2 School of Medicine, Ross University School of Medicine, Miramar, USA; 3 Department of Anesthesiology, Louisiana State University Health Sciences Center, Shreveport, USA; 4 Pain Medicine, Paolo Procacci Foundation, Rome, ITA

**Keywords:** selective estrogen receptor modulators, bisphosphonates, thioamides, thyroid dysfunction, bone mineral density, osteoporosis, hyperthyroidism

## Abstract

Normal thyroid hormone levels are crucial for the homeostasis of many metabolic cycles and processes throughout the human body. Thyroid dysfunction, such as thyrotoxicosis, can result from many different etiologies, including Graves’ disease (GD), toxic multinodular goiter (MNG), and toxic adenoma. These hyperthyroid disease states can cause devastating complications and disease, including the disruption of the bone remodeling cycle and skeletal development, which can result in osteoporosis. Osteoporosis is characterized by a decrease in bone mineral density and a propensity for fragility fractures. In addition to patients with overt hyperthyroidism, studies have provided evidence of other high-risk patient demographics, such as individuals with subclinical hyperthyroidism and postmenopausal women, who may be at an increased risk for the development of secondary osteoporosis. The treatment of patients with hyperthyroid-induced osteoporosis often requires a multifaceted management plan that may be unique to each patient’s situation. Antithyroid therapy is often the first step in treating this disease and may include thioamide medications. Radioactive iodine-131 therapy (RAI) and the surgical removal of the thyroid gland may also be reasonable approaches for restoring normal thyroid function. Following thyrotoxicosis mitigation, antiresorptive drugs such as bisphosphonates, calcitonin, and selective estrogen receptor modulators (SERMs) may be used to counteract decreased bone mineral density (BMD). Additionally, the implementation of vitamin D, calcium supplements, and weight-bearing exercise may also reduce bone loss. While the effects of thyroid stimulating hormone (TSH) and triiodothyronine (T3) on bone remodeling have been studied in the past, more research is needed to identify unknown mechanisms and develop future improved treatments for this condition.

## Introduction and background

Hyperthyroidism affects over 1% of the United States population and can result in elevated thyroid hormone release, otherwise known as thyrotoxicosis [1,2]. Hyperthyroidism can be classified as overt or subclinical, with subclinical eventually progressing to overtly in certain clinical situations [3,4]. Subclinical hyperthyroidism consists of low thyroid-stimulating hormone (TSH) and thyroid hormone (triiodothyronine (T3) and thyroxine (T4)) levels within the normal range [5,6]. Overt hyperthyroidism is characterized by suppressed TSH levels and elevated T3 and T4 levels [2,3]. Hyperthyroidism has various disease etiologies, including Graves’ disease (GD), toxic multinodular goiter (MNG), toxic adenoma, iodine-induced hyperthyroidism, and many others [3,5,7]. Depending on the etiology, treatments for hyperthyroidism range from surgical intervention to radioactive iodine-131 therapy (RAI) and antithyroid drugs [3,7].

Both overt and subclinical hyperthyroidism affect many of the body’s organ systems and are associated with long-term complications, including tachycardia, proptosis, increased sweating, weight loss, and many others [7,8]. Treatment is almost always indicated for overt hyperthyroidism and is sometimes indicated for subclinical hyperthyroidism if certain criteria are met [3]. One potential detrimental condition caused by increased thyroid hormone levels is osteoporosis [9,10]. Osteoporosis is the most common global bone disorder characterized by decreased bone mass, increased fracture incidence, and much higher morbidity and mortality rates [11-13]. Even though osteoporosis predominantly affects older women and men, secondary osteoporosis can also affect younger individuals [14]. Osteoporosis is diagnosed in individuals with a bone mineral density (BMD) of 2.5 standard deviations less than the average young adult [15,16].

To sufficiently manage osteoporosis in individuals with hyperthyroidism, it is important to understand the underlying mechanisms of the disease, diagnostic approaches, and treatment options available [9,16]. Bone metabolic activity and development depend on thyroid hormone levels, and elevated thyroid hormones without intervention may result in expedited bone development and increased bone age [9,17,18]. Suppressed TSH levels due to hyperthyroidism or persistent thyroid hormone replacement therapy have also been correlated with a higher risk of vertebral and femur fractures due to normal TSH levels acting as a negative regulator for bone remodeling [18-20]. While hyperthyroidism results in bone loss in all skeletal areas, it is most commonly associated with cortical rather than trabecular bone loss, and solely correcting thyroid function can often be sufficient to prevent excessive bone resorption [21,22]. However, the most effective treatment options may involve traditional osteoporosis management, such as vitamin D, supplemental calcium, and antiresorptive and antithyroid agents [14,21,22].

The present investigation, therefore, aims to describe the mechanism behind osteoporosis secondary to hyperthyroidism and to analyze current potential treatment options that will best improve patient outcomes. In this review, we will discuss the various etiologies and classifications of hyperthyroidism, the relationship between hyperthyroidism and osteoporosis, current options for disease treatment, clinical considerations, and potential future directions for the management of hyperthyroidism-induced osteoporosis.

## Review

Common hyperthyroidism etiologies and specific disease mechanisms

GD

GD is an autoimmune condition that primarily affects the thyroid gland and is the most common cause of hyperthyroidism [23,24]. GD can occur in various age groups; however, individuals between the ages of 20 and 50 are predominately affected [23-25]. GD is also disproportionately diagnosed in people with other autoimmune disorders, women, and individuals who smoke tobacco [23,26]. The pathophysiology of GD is delineated by the binding of autoantibodies to thyroid-stimulating hormone receptors (TSHR). These antibodies act as TSHR agonists, which cause excess secretion of thyroid hormones and independent functioning of the thyroid gland from the pituitary gland [27-29]. TSHR autoantibodies play a role in Graves' orbitopathy (GO), thyroid gland hyperplasia, pretibial myxedema, and other disease states [28,30,31]. Treatment for GD may include RAI, antithyroid medication, or even total thyroidectomy [23].

Toxic MNG

Toxic MNG is another prevalent thyroid gland disorder partially attributed to the diverse genetic variability of follicular cells and the incidence of new heritable cellular traits [32]. Toxic MNG typically occurs if nontoxic MNG is left untreated for a long duration, and multiple autonomous nodules on the thyroid gland characterize it. These nodules produce an excess number of thyroid hormones, which ultimately results in hyperthyroid states [33,34]. A nodular goiter can be identified and diagnosed by physical examination as a protruding mass in the neck, which may cause discomfort due to excess pressure on nearby anatomical structures [32,35]. While most goiters are benign and show no symptoms, they still can raise concerns due to their compressive effects on the trachea and other structures secondary to the thyroid gland enlargement [32]. The etiology of MNG is known to be multifactorial; however, iodine deficiency often plays a significant role in the development of this disorder [36,37]. Without sufficient iodine levels, thyroid hormone production decreases, causing increased TSH release, which can lead to thyroid gland hyperplasia [37]. Chronic hyperthyroidism secondary to autoimmune disease and the administration of exogenous iodine in Jod-Basedow syndrome can also result in a significant portion of MNG cases [38-40]. For healthcare providers, the diagnostic assessment of patients with this condition should involve a clinical evaluation, laboratory testing for thyroid hormone levels, and the consideration of imaging if there are further indications of any complicating factors, such as malignancy [7,23,41]. In the case of a non-toxic MNG deemed asymptomatic, options such as suppression therapy, regular clinical examinations, and monitoring TSH levels are considered appropriate clinical management [41,42]. Treatment for toxic MNG should include potential thyrotoxicosis mitigation followed by considering RAI or surgical resection [43,44]. Surgery is often recommended if compressive symptoms, risk of malignancy, or cosmetic concerns arise [41,45].

Toxic Thyroid Adenomas

Toxic thyroid adenomas are usually sporadic lesions found in the thyroid gland that actively excrete excess thyroid hormones. Still, they can also be caused by environmental factors such as iodine deficiency [46,47]. They may result from genetic mutations in the *BRAF* gene or translocation events of the *PAX8-PPAR* fusion gene [48,49]. Thyroid adenomas can be categorized as both functional and nonfunctional [46]. Mutation in the TSH receptor causing monoclonal thyroid cell expansion is the most common gene mutation associated with functional thyroid adenomas [46,50,51]. Non-functional adenomas and thyroid carcinomas typically result from mutations in the *KRAS* gene [52,53]. Adequate clinical management for asymptomatic patients includes needle aspiration and monitoring with ultrasonography, while patients experiencing symptoms may undergo surgical resection or antithyroid drug therapy [54-57].

Normal thyroid function and disease-specific mechanisms related to bone remodeling

Thyroid hormones operate via the hypothalamic-pituitary-thyroid axis (HPA), which involves the thyroid, anterior pituitary, and hypothalamus. T4 and T3 are the main hormones produced by the thyroid gland [38,40]. Iodine, obtained from sources like iodized salt, seafood, and vegetables, is crucial for T3 and T4 synthesis. Inadequate iodine intake can lead to iodine deficiency and associated conditions such as cretinism, goiter, myxedema coma, and hypothyroidism [58,59]. Any deviation from euthyroid levels can result in significant disease and dysfunction.

Thyroid hormones play an essential role in regulating metabolism and cellular processes throughout the human body. Their receptors are found in various tissues, including the nervous system, pituitary gland, lungs, heart, liver, muscle, bones, testis, and placenta [58]. Consequently, any potential changes in thyroid hormone levels may disrupt various processes throughout the body [60]. Symptoms of hyperthyroidism may include weight loss, heat intolerance, diarrhea, fine tremor, and muscle weakness [7]. One dangerous effect of elevated thyroid hormone levels is the impact on bone metabolism, often leading to a drastic reduction in bone mineral density. This condition, known as osteoporosis, is associated with the increased potential for fragility fractures and higher mortality rates [9,61]. The effects of hyperthyroidism on bone metabolism have been well documented; however, there is a severe lack of consensus in determining the effects of hypothyroidism and subclinical hypothyroidism on bone health [15].

The physiological function of thyroid hormones is necessary for normal skeletal development. Regular function and release of TSH are imperative for bone metabolism, with defects in TSH release being correlated with an increased risk for osteoporotic fractures [62]. Although there has been conflicting evidence regarding the effects of TSH on osteoblastic activity, TSH has been proposed as a crucial inhibitor of bone turnover [63,64]. TSH has been shown to directly inhibit osteoclastic bone resorption by reducing the local production of tumor necrosis factor-alpha [9]. Additionally, a nuclear T3 receptor has been discovered in both osteoblastic and osteoclastic cell lines. Studies have provided evidence that T3 may directly stimulate bone resorption with the involvement of interleukin (IL) 6, which enhances osteoclast activity [63]. However, it is still unclear whether this increase in osteoclast activity is a direct effect of T3 or if this is the result of T3-induced osteoblast activation [18]. Studies involving mutant mice have demonstrated T3 regulating bone metabolism primarily by binding to the nuclear thyroid receptor alpha (TRα). Evidence of anabolic processes stimulated by T3 during the adolescent growth phase also allows for peak bone mass accrual [65]. Overall, studies have provided evidence for the effects of T3 and TSH on bone metabolism, but some specific mechanisms remain inconclusive and require further investigation [66].

**Figure 1:**
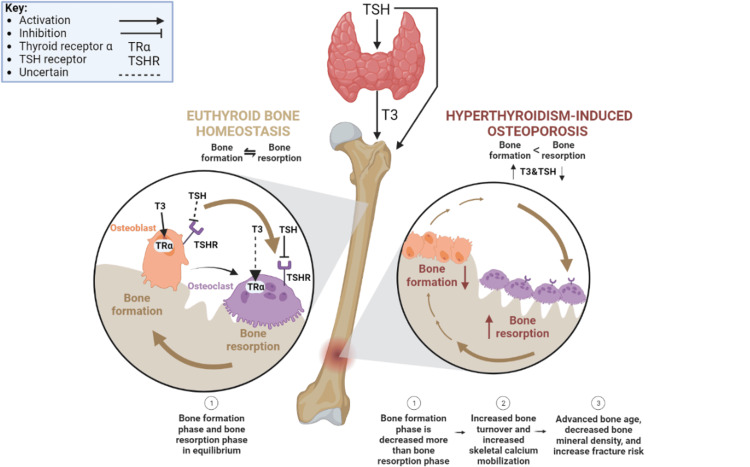
Metastasis to Bone Disrupts Bone Homeostasis Retrieved from: https://app.biorender.com/biorender-templates TSH: thyroid stimulating hormone; TRα: thyroid receptor alpha; TSHR: thyroid-stimulating hormone receptor;

Osteoporosis can be induced by increased thyroid hormone function, which may result in an imbalance between bone resorption or breakdown and bone formation. During euthyroid states, bone resorption and formation phases of the bone turnover cycle are relatively balanced, with little mineral composition and density fluctuation [67]. However, with increased T3 and decreased TSH levels, hyperthyroid states result in a shorter remodeling cycle with more bone resorption occurring than bone formation [68]. High levels of thyroid hormones in adult patients result in increased bone turnover, decreased mineral density, and a higher propensity for fracture [21]. The bone remodeling cycle involves both the T3 nuclear TRα and the TSH G-protein coupled receptors on both osteoblasts and osteoclasts (Figure 1) [68-70]. T3 has been demonstrated to cause osteoblast growth and bone formation; however, it is unknown whether T3 acts directly on osteoclasts or if its effects on osteoblasts indirectly stimulate osteoclastic activity [68]. With hyperthyroid-induced osteoporosis, the bone remodeling cycle proceeds with shorter duration and increased incidence, resulting in relatively longer bone resorption than bone formation phases [68].

Clinical considerations of patients suffering from hyperthyroid-induced osteoporosis

In the clinical setting, BMD measurements are typically taken from the distal forearm, spine, and hip of high-risk patients exhibiting osteoporotic symptoms [16]. Osteoporosis secondary to thyrotoxicosis disproportionately affects postmenopausal women to a greater extent and often results in patients presenting with bone fractures [19,71]. Historically, hyperthyroid-induced osteoporosis has been clinically diagnosed in patients with abnormally high circulating T3 and T4 levels. However, more recent studies have shown that patients exhibiting normal thyroid hormone levels and decreased TSH levels may also be at risk for the development of secondary osteoporosis [16,72,73]. These findings demonstrate the possibility of secondary osteoporosis developing from overt hyperthyroidism and subclinical hyperthyroidism.

Since hyperthyroidism causes an increase in bone metabolic activity, this can result in bone resorption occurring at a higher rate than bone formation [16,74]. While this causes an overall decrease in BMD, distinct regions and types of bone are affected to varying degrees. Cortical bone is typically affected more significantly, causing more drastic reductions in BMD than trabecular bone [75,76]. It has been demonstrated that patients suffering from hyperthyroidism-induced osteoporosis exhibited up to a 40% decrease in BMD of cortical bone in comparison to only a 2.7% decrease in BMD of trabecular bone [75]. A more recent study, including middle-aged women with hyperthyroidism, showed a statistically significant correlation between low TSH and low BMD in cortical bone. This study further illustrates the risk of patients with subclinical hyperthyroidism for developing osteoporosis and supports the idea that cortical bone is most severely affected [77].

The severity of thyrotoxicosis also contributes to overall morbidity in patients suffering from secondary osteoporosis [16]. A statistically significant decrease in BMD was observed in patients with a serum TSH level less than 0.50 mU/L [77]. This relationship between low TSH and low BMD was exhibited to the greatest extent in patients with serum TSH levels below 0.10 mU/L, demonstrating that the degree of hyperthyroidism itself can determine the overall morbidity of secondary osteoporosis [77]. In a separate prospective cohort, a serum TSH level of less than 0.10 mU/L resulted in a greater than four-fold increase in the risk of vertebral osteoporotic fractures and a greater than three-fold increase in the risk of hip fractures [20]. Higher levels of serum TSH, such as those found in the normal reference range (0.5-5.5 mU/L), were associated with a drastically decreased risk of fracture when compared to the low TSH group (<0.10 mU/L) [20].

High serum calcium levels, seen in about 8% of hyperthyroid patients, are another risk factor increasing the clinical morbidity of secondary osteoporosis [78,79]. This mechanism results from the effects of calcium levels on parathyroid hormone (PTH) secretion. High levels of serum calcium result in decreased PTH release, which reduces vitamin D conversion to its active form in the kidneys [79,80]. This decrease in active vitamin D causes further reductions in BMD, leading to increased morbidity of osteoporosis in these patients [16].

Overall, the investigation of many studies provides evidence that the degree of hyperthyroidism is one of the most significant factors contributing to morbidity associated with hyperthyroid-induced osteoporosis. Furthermore, high-risk patient groups such as post-menopausal women and individuals with subclinical hyperthyroidism should be considered for osteoporotic screening and prophylactic treatment to mitigate adverse outcomes. Prospective studies less than 10 years old that analyze the effects of thyroid hormones on bone metabolism were obtained from PubMed and included in Table 1.

**Table 1:** Comparative Studies Analyzing the Effects of Thyroid Hormones on Bone Metabolism HR: hazard ratio; TSH: thyroid stimulating hormone; GD: Grave’s disease; MNG: multinodular goiter; BMD: bone mineral density

Study	Groups Studied and Intervention	Results and Findings	Conclusions
Study 1: Bloom et al., 2015 [81]	Thyroid function and subsequent fracture data from 13 prospective cohort studies, including 70,298 participants, were analyzed.	After adjustment for other risk factors, endogenous subclinical hyperthyroidism was associated with a HR of 1.42 (95%CI, 1.16-1.74) for any fracture. TSH level < 0.10 mlU/L was associated with the highest hip, spine, and non-spine fracture risk.	Individuals suffering from subclinical hyperthyroidism are at an increased risk of fracture.
Study 2: El Hadidy et al., 2011 [82]	This study included 52 male subjects with hyperthyroidism secondary to GD or toxic MNG, along with 25 controls. Biochemical assays indicating bone turnover were obtained, including serum total calcium, phosphorus, total alkaline phosphatase, bone-specific alkaline phosphatase (B-ALP), osteocalcin (OC), and carboxy-terminal telopeptide of type l collagen (β-CTx). Urinary calcium, urinary deoxypyridinoline (DXP), and urinary creatinine were also collected.	Bone turnover serum markers were significantly higher in patients with GD and toxic MNG than in controls (P < 0.01). Bone density was considerably lower in GD and toxic MNG compared to the control group. Free T3 and free T4 were positively correlated with the collected biochemical markers of bone turnover.	Men with hyperthyroidism have significant bone loss and increased biochemical markers of bone turnover. The hyperthyroid state's severity and duration directly correlate with the degree of bone turnover and loss in these individuals.
Study 3: Daya et al., 2022 [83]	10,946 patients were included from the prospective study Atherosclerosis Risk in Communities (ARIC), which began in 1987. Thyroid function was analyzed from nine different visits, with the 9^th^ still occurring at the time of study publication. The study's hospital surveillance and access to the Centers for Medicare and Medicaid Services (CMS) databases captured subclinical thyroid dysfunction and the primary outcome of fracture risk.	The adjusted HR of incident fractures in individuals with subclinical hyperthyroidism was 1.34 (95% CI, 1.09-1.65). Individuals with normal T4 levels were more highly associated with fracture-related hospitalization if they presented with thyrotropin concentrations below 0.56 mIU/L.	Evidence for subclinical hyperthyroidism being a risk factor for the fracture incident was demonstrated.
Study 4: Waring et al., 2013 [84]	Data was gathered from a study consisting of men over 65 years old. Baseline serum samples were collected from 397 men with non-spine fractures and 1420 men without fractures. Thyrotropin (TSH) and free thyroxine (FT4) levels were measured. Bone density was assessed using hip dual-energy X-ray absorptiometry (DXA). Follow-up was conducted after approximately 4.6 years of fracture risk, and bone loss was evaluated.	After adjustment, thyrotropin (TSH) levels were not associated with an increased risk of non-spine fractures (relative hazard (RH) 0.92 per standard deviation decrease in TSH (95%CI, 0.74-1.14)). However, there was a significant correlation between TSH levels and the risk of hip fractures (RH 1.31; 95%CI, 1.01-1.71), even within the normal range of TSH values (RH 1.21; 95% CI, 1.00-1.47). There was no correlation between TSH or FT4 levels and bone loss, and the risk of fractures did not significantly differ across different thyroid function categories.	TSH and FT4 levels are not linked to bone loss, but it should be noted that lower serum TSH levels may be associated with a higher risk of hip fractures in older men.
Study 5: Deng et al., 2021 [70]	This study analyzed BMD, free thyroxine (FT4), free triiodothyronine (FT3), and TSH levels in 114 men in euthyroid states. Rat osteoblasts were also exposed to different levels of TSH to observe its effects on gene and protein expression.	TSH levels were demonstrated to be positively correlated with BMD. TSH levels at 10 mU/mL and 100 mU/mL resulted in a significant upregulation of genes relating to osteoblast function. Additionally, bone morphogenetic protein two activity increased with higher TSH levels over extended periods.	TSH concentration and BMD were positively correlated in males with normal thyroid function. TSH was also shown to increase rat osteoblast differentiation and proliferation.

Current treatment options for secondary osteoporosis

Osteoporosis induced by hyperthyroidism is a complex condition that requires sufficient management to prevent bone loss and reduce the risk of fractures [1,2]. The first crucial step in management is addressing the underlying hyperthyroidism. The thyroid hormones T4 and T3 regulate various physiological processes, including metabolism, growth, and development [2,9]. However, an overactive thyroid gland can disrupt bone metabolism. Treatment options for hyperthyroidism include antithyroid medications, radioactive iodine therapy, and surgery [1-3].

Thioamides

The thioamide medications, methimazole, carbimazole, and propylthiouracil (PTU) are thyroid antagonists that exert their therapeutic effects by impeding the synthesis and secretion of thyroid hormones [1-3]. The mechanism of PTU involves the inhibition of the enzyme thyroid peroxidase, which typically converts iodide into its active form and integrates it with tyrosine [2,85]. Consequently, the production of crucial components used in the synthesis of thyroid hormones, diiodotyrosine (DIT) or monoiodotryosine (MIT), is also prevented [85]. Furthermore, PTU obstructs the peripheral conversion of T4 to T3 and interferes with stored thyroid hormones that have already been synthesized [2,3,85]. Methimazole, and its prodrug carbimazole, hinder the process of the iodination of tyrosine residues in thyroglobulin, which is facilitated by the thyroid peroxidase enzyme. As a result, the production of T4 and T3 is blocked [2,3,86]. However, this medication does not impact synthesized thyroxine and triiodothyronine already present in the bloodstream or thyroid gland [3,86].

Dosing for thioamides is titrated based on thyroid hormone levels and the clinical manifestation of the patient [1,85,86]. Achieving a euthyroid state can take several months following the initiation of therapy. While methimazole and PTU are equally effective, methimazole is favored due to its once-daily dosing and relatively better safety profile, except for pregnant patients, for whom PTU is preferred [3,85-88]. The severe side effects of PTU and methimazole include liver injury and agranulocytosis [1,85,86,88]. Regular monitoring of thyroid function tests, accompanied by patient education to promote awareness of potentially serious side effects, is essential for effectively maintaining normal thyroid function [1,3,85,86].

RAI

RAI is often the preferred therapy for patients, especially those with high-risk comorbidities who are unsuitable for surgery and require definitive management [1,3]. A single oral dose of radioactive iodine is administered and absorbed by the thyroid gland. It induces an inflammatory reaction within thyroid follicular cells, resulting in thyroid fibrosis and gradual destruction over the following months. Hypothyroidism typically develops within 6-12 months, making lifelong hormone replacement therapy necessary for most patients [2,3]. RAI is also recommended for patients not able to take thioamide medication. However, caution should be exercised for patients planning on being pregnant or breastfeeding within six months due to the risk of hypothyroidism in the fetus [3]. RAI is also contraindicated in patients with moderate to severe GO or underlying thyroid malignancies [2,3]. Thioamide use should be stopped approximately one week before RAI therapy, and patient monitoring is required as it may take several months to achieve normal thyroid function following initial RAI treatment [3].

Thyroidectomy

In cases where medication or RAI is not practical or contraindicated, surgical intervention may be necessary [3,89]. Thyroidectomy is a surgical procedure that involves surgically removing either a portion or the entirety of the thyroid gland. After undergoing a significant or complete thyroidectomy, patients should begin weight-adjusted levothyroxine replacement therapy (0.8 mcg per pound or 1.6 mcg per kilogram) to maintain euthyroid levels [3].

Bisphosphonates

Once the patient is undergoing hyperthyroidism treatment, the next step is to counterbalance its harmful effects on BMD [90]. Bisphosphonates are a class of medications frequently utilized for the treatment of osteoporosis that inhibit bone resorption and preserve bone density [16,91]. Bisphosphonates are pyrophosphate analogs that bind hydroxyapatite crystals in sites of active bone remodeling [91]. Nitrogen-containing bisphosphonates commonly used in clinical practice include alendronate, risedronate, and zoledronic acid. These drugs inhibit farnesyl pyrophosphate synthase, part of the cellular pathway involving the modification of small GTP-binding proteins [16,91-93]. The osteoclasts affected will undergo apoptosis with this essential pathway inhibited [91-93]. Bisphosphonates reduce bone resorption and help maintain bone density, decreasing fracture risk [91-94].

Calcitonin

Calcitonin, a hormone primarily secreted by the parafollicular cells of the thyroid gland, is vital in regulating calcium levels within the body [95]. It primarily influences osteoclasts and the kidney's tubular epithelium. Its action within the kidney promotes diuresis and reduces calcium and phosphate reabsorption, resulting in lower serum levels of both. Calcitonin also induces the contraction of osteoclasts and diminishes their capacity to degrade bone tissue. Furthermore, it alters the ideal acidic conditions necessary for osteoclast activity by inhibiting carbonic anhydrase II and impedes osteoclast precursors' maturation [95]. Although calcitonin results in decreased bone resorption and serum calcium levels, it is essential to consider that its efficacy dwindles after one to two days [95].

Selective Estrogen Receptor Modulators (SERMs)

SERMs are another group of medications that can be used as an adjunct for treating osteoporotic postmenopausal women with hyperthyroidism [16,96,97]. The most common SERMs include bazedoxifene and raloxifene. Current hypotheses propose various mechanisms by which SERMs act as estrogen agonists that modulate bone regulation and remodeling [96,97]. These mechanisms include potential reductions in bone-degrading cell formation (osteoclastogenesis) through mitochondrial and estrogen receptor (ER)-mediated pathways [96,97]. Additionally, bazedoxifene may also play a role in regulating serum calcium levels [96,97].

Monoclonal Antibodies and Remodeling Agents

Other drug therapies may be useful in treating secondary osteoporosis, including denosumab and teriparatide. Denosumab is a monoclonal antibody that prevents osteoclast RANK receptor activation, thus inhibiting bone resorption [98]. Studies have shown that denosumab effectively increases bone mineral density, reduces the risk of fragility fracture, and decreases bone turnover markers in the serum [99,100]. However, discontinuation of denosumab may be associated with an increased incidence of multiple vertebral fractures due to the sudden loss of RANK receptor inhibition and rebound bone density loss [101]. Other anti-resorptive drugs, such as bisphosphonates, are often used to combat these negative effects of discontinuation [98]. Remodeling agents such as teriparatide may be more suitable for patients who are unable to tolerate bisphosphonate therapy due to contraindication, but studies have shown that this switch may result in significant and progressive bone loss [98,102].

Teriparatide is a PTH analog that mimics the actions of PTH by increasing serum calcium levels and decreasing phosphate absorption in the kidney [103]. Similar to PTH, teriparatide also increases the activity of both osteoblasts and osteoclasts, leading to increased bone growth and osteoclastic differentiation [104]. Teriparatide has been shown to increase BMD in patients with a high risk for osteoporotic fractures and may promote recovery following osteonecrosis of the jaw secondary to long-term bisphosphonate therapy [104,105]. While teriparatide may be an effective treatment for secondary osteoporosis, it is often only recommended for patients at high risk for fragility fracture due to its possible association with increased incidence of osteosarcoma, role in calcium and phosphate metabolism, limited availability through injection, and cost [103,106].

Lifestyle Modification, Calcium, and Vitamin D

In addition to these pharmacological interventions, lifestyle modifications are essential in managing hyperthyroidism-related osteoporosis. Regular physical activity, particularly weight-bearing exercises, has been proven beneficial for bone health [19]. Weight-bearing exercises involve activities in which the body supports its weight against gravity, such as walking, jogging, dancing, or strength training [107]. These exercises stimulate bone remodeling and help maintain BMD [108,109]. Calcium and vitamin D supplementation are crucial in maintaining bone health and preventing further bone loss [11,16]. Calcium is a fundamental mineral for bone structure and strength, and vitamin D aids in the absorption and utilization of calcium [94]. Adequate intake of these nutrients is essential, and supplementation is often recommended if dietary intake is insufficient [110].

Osteoporosis induced by hyperthyroidism requires a multifaceted approach for effective management. Treating the patient’s underlying hyperthyroidism is crucial, and interventions such as antithyroid medications, radioactive iodine therapy, or surgery can help to manage thyroid hormone levels. Antithyroid treatment in combination with calcium and vitamin D supplementation, bisphosphonates, calcitonin, and SERMs helps to prevent further bone loss, promotes bone formation, and reduces fracture risk [16,90]. Incorporating lifestyle modifications and engaging in weight-bearing exercises synergistically complements the effectiveness of these medical interventions [107-109]. Articles discussing respective drug mechanisms that have been updated within the past year were obtained from PubMed and included in Table 2.

**Table 2:** Antithyroid and Antiresorptive Drug Mechanisms, Indications, and Considerations MNG: multinodular goiter; SERM: selective estrogen receptor modulator

Drug	Mechanism of Action	Indications	Considerations
Propylthiouracil [85]	Inhibits thyroid peroxidase and peripheral conversation of T4 to T3	Hyperthyroidism, Graves’ disease, Toxic MNG, Pre-treatment for thyroidectomy or radioactive iodine therapy, Thyrotoxicosis crisis,	Not recommended for pediatric use due to liver injury risk, not used in patients with liver impairment, Preferred antithyroid drug in the first trimester of pregnancy, use methimazole in second and third trimesters, excreted in breast milk, no clear recommendations for use during breastfeeding, caution in geriatric patients
Methimazole [86]	Inhibits thyroid peroxidase and iodotyrosyl residue coupling	Graves’ disease, Toxic MNG, Pre-treatment for thyroidectomy or radioactive iodine therapy, thyrotoxicosis crisis	Excreted in breast milk, contraindicated during pregnancy as it is a category D drug capable of fetal harm
Bisphosphonates [93]	Inhibit osteoclast activity by inhibiting farnesyl pyrophosphate synthase	Osteoporosis prophylaxis and treatment	Contraindicated for patients with a history of hypocalcemia, chronic kidney disease, achalasia, esophageal varices, atypical femur fracture due to bisphosphonates and osteonecrosis secondary to bisphosphonates
Bazedoxefene [96]	SERM, which modulates bone metabolism most likely by reducing osteoclast activity and altering calcium levels	Primarily used for osteoporosis management in postmenopausal women, Prophylaxis for glucocorticoid-induced osteoporosis	Contraindicated for pregnancy and individuals with a history of deep vein thrombosis, stroke, pulmonary embolism, or myocardial infarction
Raloxifene [97]	SERM binds estrogen receptors and has an agonistic effect on bone, which decreases bone resorption while increasing BMD	Treatment and prophylaxis for osteoporosis, used for reducing the risk of metastatic breast cancer	Contraindicated for pregnancy and individuals with a history of deep vein thrombosis, renal vein thrombosis, pulmonary embolism, stroke, myocardial infarction, malignancy, smoking, or thrombophilia

Discussion

Normal thyroid hormone levels are essential for the growth and development of the human body, with fluctuations causing severe dysfunction [6]. Hyperthyroidism, often resulting from GD, toxic MNG, or toxic adenoma, affects most organ systems and metabolic processes throughout the body [23,33,46,58]. The importance of understanding the mechanisms and disease states secondary to hyperthyroidism is evident due to its prevalence in the United States being approximately 1.2% [1]. By appropriately managing hyperthyroidism, negative secondary outcomes, such as hyperthyroidism-induced osteoporosis, may be reduced significantly [21].

In addition to overt hyperthyroidism, studies have demonstrated that postmenopausal women and individuals with subclinical hyperthyroidism are at a higher risk for developing secondary osteoporosis [16,72,73]. As patients with osteoporosis are more likely to suffer from increased mortality rates and risk of fracture, future studies must investigate the efficacy of current treatments and the development of improved therapies for this patient population [9,61]. While it is commonly known that increased T3 and decreased TSH levels result in a shorter bone remodeling cycle favoring resorption, more studies are needed to delineate the effects of TSH on osteoblasts and T3 on osteoclasts [64,68,70]. However, studies have demonstrated the inhibitory effects of TSH on osteoclasts and the stimulation of osteoblasts by T3. This provides insight into the development of decreased bone mineral density and increased fracture risk in individuals suffering from hyperthyroidism [64,68].

Hyperthyroid-induced osteoporosis has been shown to predominately affect cortical bone rather than trabecular bone [75,76]. Furthermore, the morbidity of secondary osteoporosis has been demonstrated to directly correlate with the severity of hyperthyroidism and hypercalcemia [16,78,79]. As TSH levels drop with increased thyrotoxicosis severity, bone mineral density levels significantly decrease, resulting in an increased risk of vertebral, hip, and other fractures [20,77].

Diverse etiologies and disease states resulting in thyrotoxicosis cause the management of secondary osteoporosis in each patient challenging and unique. The first step in reducing the morbidity of hyperthyroid-induced osteoporosis is controlling the patient’s thyroid hormone levels [2]. Antithyroid therapies such as methimazole, propylthiouracil, radioactive iodine therapy, and thyroidectomy are all viable options for maintaining appropriate thyroid hormone levels in patients suffering from thyrotoxicosis [3,89]. Proper thyroid management is followed by osteoporosis mitigation, which may include antiresorptive medications such as bisphosphonates, calcitonin, and selective androgen receptor modulators [16,91,95,96]. In addition to drug therapy, weight-bearing physical activity and routine calcium and vitamin D supplementation are recommended to improve bone mineral density further [11,16,19].

## Conclusions

Antithyroid and secondary osteoporosis management is essential when dealing with high-risk patient demographics, such as individuals with overt hyperthyroidism, men with subclinical hyperthyroidism, and postmenopausal women. Many useful treatment options have been studied and utilized to manage hyperthyroid-induced osteoporosis, ultimately reducing disease morbidity and mortality. However, further research is needed to identify the unknown specific mechanisms of hyperthyroid-induced osteoporosis, analyze the efficacy of current treatment options, and possibly develop new drugs and therapies for managing this disease.
